# Label-Based Alignment Multi-Source Domain Adaptation for Cross-Subject EEG Fatigue Mental State Evaluation

**DOI:** 10.3389/fnhum.2021.706270

**Published:** 2021-10-01

**Authors:** Yue Zhao, Guojun Dai, Gianluca Borghini, Jiaming Zhang, Xiufeng Li, Zhenyan Zhang, Pietro Aricò, Gianluca Di Flumeri, Fabio Babiloni, Hong Zeng

**Affiliations:** ^1^School of Computer Science and Technology, Hangzhou Dianzi University, Hangzhou, China; ^2^Industrial NeuroScience Lab, University of Rome “La Sapienza”, Rome, Italy; ^3^Key Laboratory of Brain Machine Collaborative Intelligence of Zhejiang Province, Hangzhou, China

**Keywords:** electroencephalogram, label-based alignment, multi-source domain adaptation, cross-subject, individual differences, fatigue mental state

## Abstract

Accurate detection of driving fatigue is helpful in significantly reducing the rate of road traffic accidents. Electroencephalogram (EEG) based methods are proven to be efficient to evaluate mental fatigue. Due to its high non-linearity, as well as significant individual differences, how to perform EEG fatigue mental state evaluation across different subjects still keeps challenging. In this study, we propose a Label-based Alignment Multi-Source Domain Adaptation (LA-MSDA) for cross-subject EEG fatigue mental state evaluation. Specifically, LA-MSDA considers the local feature distributions of relevant labels between different domains, which efficiently eliminates the negative impact of significant individual differences by aligning label-based feature distributions. In addition, the strategy of global optimization is introduced to address the classifier confusion decision boundary issues and improve the generalization ability of LA-MSDA. Experimental results show LA-MSDA can achieve remarkable results on EEG-based fatigue mental state evaluation across subjects, which is expected to have wide application prospects in practical brain-computer interaction (BCI), such as online monitoring of driver fatigue, or assisting in the development of on-board safety systems.

## 1. Introduction

Mental fatigue is incrementally formed by long-time tedious tasks, which is related to a drastic decrease in alertness (Maglione et al., 2014; Charbonnier et al., 2016). Electroencephalogram (EEG) records the complex neurophysiological activities from the cerebral cortex, which can directly reflect the potential mental state of subjects. Due to the characteristics of noninvasiveness, portability, and small cost, as well as the superiority of machine learning (ML) or deep learning (DL) in feature extraction and classification from a large amount of data, EEG-based methods by ML or DL have attracted more and more attention during recent decades (Kong et al., 2017; Monteiro et al., 2019). Nevertheless, there are still some challenges since EEG has significant differences across subjects, mainly caused by either physical (e.g., environment and skin-electrode impedance) or biological (e.g., differences in gender, age, and brain activity patterns) factors (Subha et al., 2010). The methods of traditional EEG-based analysis generally assume the data of training and testing shares the same feature distribution (Wan et al., 2021), and most methods evaluate the mental states for intra- or inter-subject [intra-subject EEG evaluation is session-to-session generalization for the same subject (Li et al., 2019b), while that of inter-subject is cross-session generalization by mixing sessions from different subjects together] (Dasari et al., 2017; Xu et al., 2018). But the performance of the existing methods sometimes degrades heavily in cross-subject EEG analysis, in which cross-subject EEG evaluation is a subject-to-subject generalization (Zhang et al., 2020b), due to the significant differences (Chai et al., 2016a; Zhang et al., 2020a). Thus, it is desired to construct a universal model for cross-subject EEG analysis.

Recently, many transfer learning (TL) methods have been widely used in such fields as motor imagery classification (Zhang et al., 2021), mental fatigue recognition (Liu et al., 2020), and emotion recognition (Li et al., 2019b). TL focuses on applying the knowledge learned from one domain (source domain) into a different but related domain (target domain) (Liang and Ma, 2020). In the TL-based cross-subject EEG analysis task, the collected EEG samples are inclusive in the source domain and target domain, respectively, that is, EEG samples from some of the subjects are regarded as the source domain, and those from the other different subjects as the target domain. Based on TL, we can explore and exploit features from the source subjects to train a model and make it adaptable to a new target subject. As a main research direction of TL, unsupervised domain adaptation (UDA) algorithms have been proven to efficiently reduce the distribution gap between each domain by matching domain-invariant features (transferable features between different domains) (Saito et al., 2018). An important advantage of UDA is that, under the condition of the same or similar label categories between source and target domains, through training on labeled data in the source domain, better classification performance can be still obtained by UDA whether samples with labels in the target domain are sufficient or not. Therefore, some researchers apply UDA-based algorithms or their variations for EEG-based mental states evaluation (Zhang et al., 2021).

As a mainstream research trend, multi-source domain-based UDA methods have broad application prospects, which extract the respective domain-invariant features by mapping and aligning the features into a common feature space between each of the source domains and the target domain, and then perform decision to the target domain separately, which is called prediction decisions result (Peng et al., 2019). However, due to the inconspicuous features near the decision boundary in the target samples, the results predicted by different classifiers may be inconsistent. To address this issue, one of the common methods is to align the probability distributions of the target samples predicted by each source domain classifier, and the average of the prediction results of all source domain classifiers is regarded as the objective function to optimize, which can minimize the differences of prediction results (Zhu et al., 2019).

In addition, regarding alignment forms, UDA-based methods mainly adopt feature-based alignment. The main idea of the alignment algorithm is to perform global feature-based alignment by mapping the source domain and target domain data into a common feature space, and extracting domain-invariant features, so as to minimize domain discrepancy (Chen et al., 2019).

Due to the high non-linearity and significant individual differences of EEG, it is difficult to extract the same or similar features for different subjects with inconspicuous features (Wan et al., 2021). Therefore, the existing UDA methods have the following two aspects of limitations for cross-subject EEG analysis. Firstly, for the issue of inconspicuous features near the decision boundary, the existing models are difficult to reach the optimal state and may fall into a local optimal state. Secondly, it is also difficult to satisfy feature-based alignment and extract domain-invariant features.

Therefore, to address the above mentioned issues, we propose a Label-based Alignment Multi-source Domain Adaptation model (LA-MSDA) which includes (1) a local label-based alignment strategy, instead of feature alignment, since the categories of labels in EEG of each subject are the same when collecting through the same paradigm (e.g., in the event-related potentials (ERPs) experiment, the actions corresponding to the induced stimulations can be regarded as labels, in which ERPs represent the neural response to specific cognitive events). In this way, it will facilitate extracting label-based domain-invariant features to eliminate the negative impact of significant individual differences of EEG, (2) an improved UDA method with global optimization. For details, setting similarity weight constraints according to the prediction probability distribution results of each classifier. A global objective function optimization strategy is introduced to address the classifier confusion decision boundary issues and improve the generalization ability of LA-MSDA in cross-subject EEG analysis.

The rest of this article is arranged as follows. Section 2 is a brief review of EEG-based related work, including traditional ML and TL. In section 3, EEG data collection and preprocessing are described. Section 4 is the LA-MSDA framework, and the experiment results are shown in section 5. Section 6 discusses and analyses the results. Finally, conclusions are given in section 7.

## 2. Related Work

In recent years, various TL-based algorithms have developed in EEG signal analysis (Lotte et al., 2018). Raghu et al. (2020) attempted to classify EEG-based multi-class seizure type by applying convolutional neural network and TL. The UDA method based on subspace alignment auto-encoder was proposed to measure the complexity of EEG signals, which considered nonlinear transformation and a consistency constraint (Chai et al., 2016b). In Li et al. (2019a), the authors proposed a DA-based model to recognize EEG emotion by making the source and the target similar in the latent representations.

Recently, some research has appeared for cross-subject EEG analysis by multi-source UDA or its variations (Xu et al., 2019), which integrate multiple source classifiers to tune the target classification model. By making up for the insufficiency of new data, Liang and Ma (2020) used a multi-source fusion transfer learning (MFTL) algorithm for mental states classification, which is based on the Riemannian manifold framework to select high similarity multiple source subjects to target subjects aimed to reduce the difference of feature distribution between source and target subjects. In Li et al. (2019b), the proposed multi-source transfer model achieved fast deployment by locating appropriate sources and mapping destinations in style transfer mapping for cross-subject emotion recognition tasks, and tested it into both supervised and semi-supervised learning.

In addition, for the multi-source domain, decision-level fusion attempts to process each source domain separately, and combine the results of respective classifiers for final recognition (Huang et al., 2016). In the cross-domain classification task with multi-source domains, Zhu et al. (2019) just used the average of all source classifier outputs to predict the labels of target data. To classify EEG-based intra-dataset emotion mental states, Lan et al. (2018) also regarded the mean classification accuracy of several domain adaptation methods as the final classification accuracy of the target data.

Due to its effective optimization of complex data, feature-based alignment algorithms have been introduced to minimize the domain discrepancy (Wang and Mahadevan, 2011). For EEG data analysis, multi-subject subspace alignment (MSSA) was proposed to decrease domain discrepancy (Chai et al., 2018), which utilized subspace alignment strategy and multi-subject information in a common framework to build personalized models for EEG-based emotion recognition. He and Wu (2019) proposed Euclidean space EEG data alignment method to minimize the distance between the mean covariance matrices in different domains by transforming and aligning the EEG data in the Euclidean space.

To sum up, previous techniques for EEG-based mental states evaluation mainly focus on aligning global feature distributions to minimize the differences between each subject, or combining all source classifiers to make a final decision. However, it is still hard to extract domain-invariant features and adapt the samples with inconspicuous features near the decision boundary across subjects. Hence, we introduce a local label-based alignment strategy to extract label-based domain-invariant features. Additionally, an improved UDA method with global optimization is proposed to address the inconspicuous features near the decision boundary issue that existed in cross-subject EEG samples and improve the generalization ability of our proposed model.

## 3. Materials

### 3.1. EEG Data Collection

**Subjects**. In the experiment, the subjects recruited should be healthy without mental illness, they all need to possess a qualified manual gear driving license and have extensive driving experience. Before the experiment, the subjects should not be allowed to drink alcohol, caffeine, and tea. Each subject is informed in advance of the experimental procedure and signs a written consent form. This experiment is approved by the local ethics committee of the University of Rome Sapienza (Rome, Italy). At last, 15 healthy subjects from 23 to 25 are selected to participate in the experiment.

**Experimental protocol**. The experiment is performed between 2 p.m. and 5 p.m. in a quiet and isolated environment. To simulate real driving scenarios, the immersive driving platform uses Alfa Romeo Giulietta QV to perform driving tasks under different conditions.

**Figure 1:**
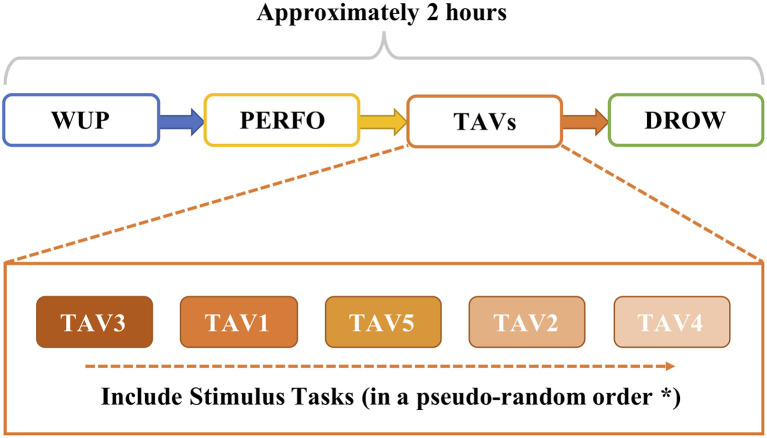
The procedure of experimental paradigm (*all the subjects will perform the task of alert and vigilance (TAV) tasks in the same order).

Table 1 describes the eight tasks of this experiment. The tasks of alert and vigilance (TAV) introduce additional video and audio to stimulate different mental states by adjusting the difficulty of driving tasks (Borghini et al., 2014; Vecchiato et al., 2016). The alert stimuli are designed with video to simulate real-world traffic jams (e.g., traffic lights, pedestrians around, other vehicles, or other uncontrollable traffic events), and a succession of frequent (with a 95% probability rate) and rare (5% probability) tones continuously delivered to the subjects as the vigilance task to simulate the noise produced during driving (e.g., car radio, engine noise, or phone celling). There are 5 stages TAV1-5 with different levels of difficulty, in which the difficulty level is increased by increasing the stimulation frequency in the simulated driving. At the beginning of the experiment, the subjects are required to drive the vehicle at a predetermined baseline speed and keep the vehicle within the lane. Such a driving condition is named as warm-up (WUP) and serves to collect the baseline for the spontaneous EEG signals in the cerebral cortex. Then, the second drive condition requires the subject to drive at a faster speed compared with WUP, named performance (PERFO). After that, the above different level task of TAVs are executed in a pseudo-random order: TAV3, TAV1 (the easiest task), TAV5 (the most difficult task), TAV2, and TAV4, which can introduce the different level of workload demands and mental states (Zeng et al., 2019). After highly stressful mental activities of TAVs, monotonous and simple tasks will make it easier for subjects to evolve into fatigue. Therefore, the driving conditions of the last task (DROWS) have no stimulation and only require driving at a fixed speed of 70 km/h. At the end of each task of the experiment, the subjects are asked to fill in the NASA-TLX questionnaire to collect the subjective information about workload perception (Hart, 2006). Furthermore, the behavioral data of subjects performing TAV tasks are also analyzed. The whole experiment process takes about 2 h or more. The flowchart of the experimental paradigm is shown in Figure 1.

**Table 1:** The details of the experiment tasks.

**Task**		**Stimuli**	**Description**
WUP (warm-up)		No	Driving the car at a baseline speed and stay in the lane, collecting the baseline of EEG signals.
PERFO (performance)		No	Requiring a faster speed compared with WUP.
TAVs (task of alert and vigilance)	TAV3	Yes (audio/ video)	Stimulating with video and audio for facilitating different mental states; There are 5 levels of TAV stages: TAV1-5, that with increasing difficulty level by setting the increase of frequency of stimulation rates in the simulated driving; The tasks of TAVs are executed in a pseudo-random order: TAV3, TAV1, TAV5, TAV2, TAV4.
	TAV1		
	TAV5		
	TAV2		
	TAV4		
DROWS		No	Setting a fixed driving speed of 70 km/h and without any stimuli.

According to the off-line analysis of the NASA-TLX and behavioral data, we choose the two mental states of TAV3 and DROWS for the subsequent analysis. As the first stage of external stimuli tasks (TAV3) with sound and video stimuli, the subjects are in a high workload state and execute the task as quickly and efficiently as possible, so the subjects are in the most awake state in TAV3. After completing a series of complex tasks, the most boring and monotonous task of DROW was finally performed without any stimuli. At this time, the workload of the subjects was the lowest, and the mental state was prone to fatigue. Hence, the collected data at TAV3 (awake with a label of 0) and DROWS (fatigue with a label of 1) are used for fatigue mental state evaluation.

### 3.2. EEG Preprocessing

**Figure 2:**
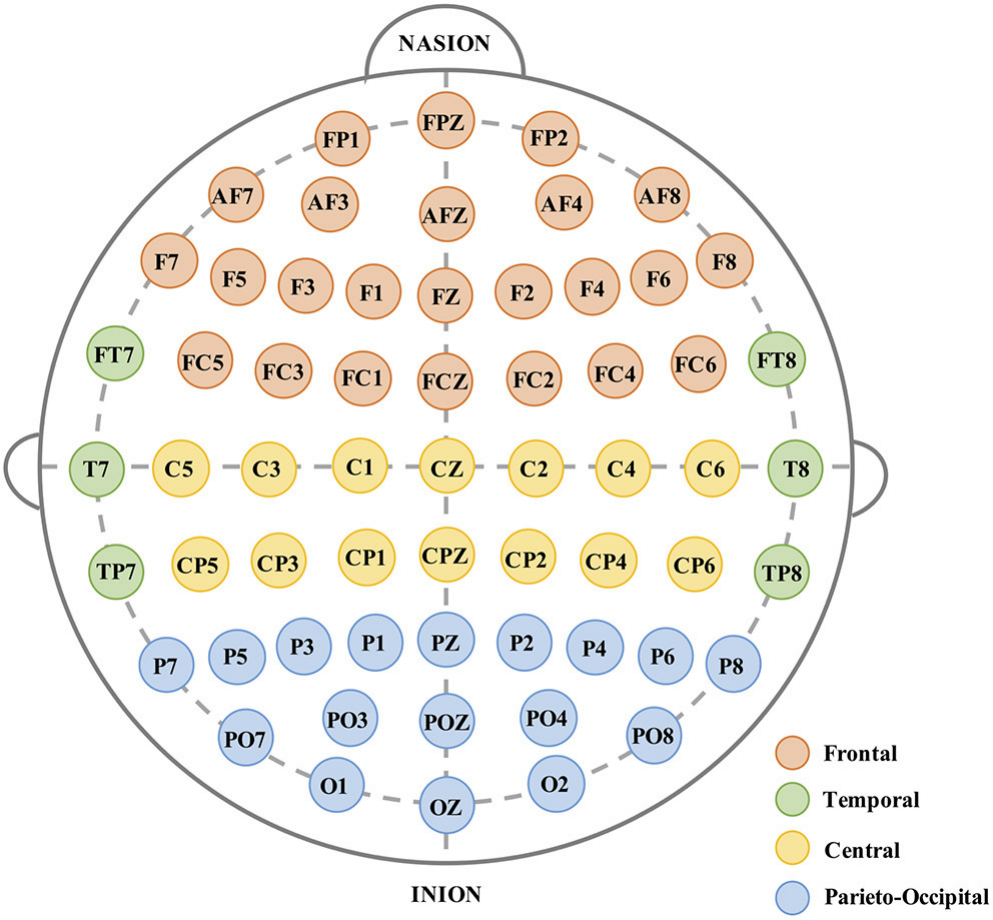
Placement of the recorded 61-channel electroencephalogram (EEG).

During the experiment, EEG data is recorded using a digital ambulatory monitoring system (Brain Products GmbH, Germany) from 61 active electrodes that are positioned according to the international 10–20 system. The 61 EEG channels recorded are (as shown in Figure 2): frontal (FP: 1, z, 2; AF: 7, 3, z, 4, 8; F: 7, 5, 3, 1, z, 2, 4, 6, 8; FC: 5, 3, 1, z, 2, 4, 6), temporal (T7, T8, FT7, FT8, TP7, TP8), central (C: 5, 3, 1, z, 2, 4, 6; CP: 5, 3, 1, z, 2, 4, 6), and parieto-occipital (P: 7, 5, 3, 1, z, 2, 4, 6, 8; PO: 7, 3, z, 4, 8; O: 1, z, 2). The data is sampled at 200 Hz. When recording the EEG data, all the electrodes are referenced to the earlobes and the impedances are below 10 (*KΩ*). To further filter the noise and remove the artifacts, the original EEG are then processed with a bandpass filter (1–30 Hz) and used Independent Component Analysis (ICA) method (Hyvärinen and Oja, 2000; Zeng et al., 2017) to remove the artifacts caused by Electrooculography (EOG), respectively.

**Figure 3:**
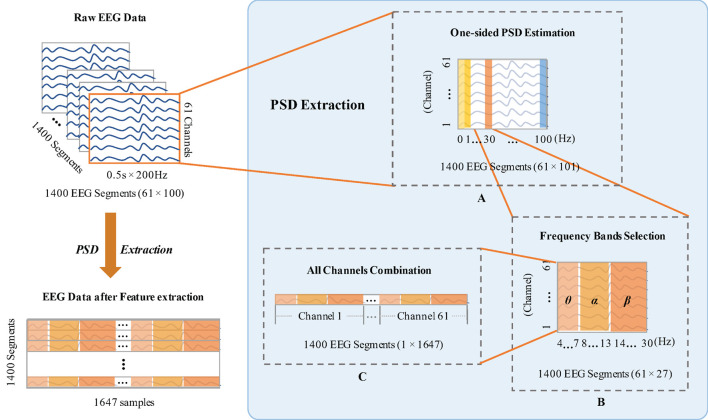
The preprocessing of each subject EEG data by power spectral density (PSD). **(A)** Introduces the one-sided PSD estimation to determine the logarithm of the signal power between 1-100Hz. **(B)** Obtains the PSD features at three selected frequency bands. **(C)** Gets 1647 characterizes from all channels in each segment.

After that, the EEG data of each channel is divided into segments with 0.5 s sliding windows without overlapping. The total number of the segments is 1,400 for each channel, including 700 segments for TAV3 and 700 segments for DROWS, respectively. Thus, in subsequent experiments, it will be conducted on 21,000 (15 × 1,400) segments of 15 subjects. Then, the EEG features are extracted from each segment. As mentioned in Bhattacharyya et al. (2014), power spectral density (PSD) is usually used to extract accurate and stable features for EEG signals analysis. Therefore, we use PSD to characterize the EEG segments, as shown in Figure 3.

Due to the 0.5 s sliding window of each channel and 200 Hz of the sampling frequency, the sample points for each window are 0.5 × 200 = 100. Thus, the feature dimension of 61 channels is 61 × 100 = 6,100. In Figure 3A, the one-sided PSD estimation is utilized to orientate the logarithm of the signal power at each point of integer frequency between 1 and 100 Hz (Martin, 2001). Existing studies have indicated that the EEG power of θ, α, and β bands can reflect the differences when human mental states change. It has been previously noted that EEG spectral power increased in θ (4–7 Hz) band could be correlated with the occurrence of mental fatigue (Borghini et al., 2012). α (8–13 Hz) band has been suggested to characterize fatigue when compared with the normal mental states (Simon et al., 2011). The mean power in β (14-30 Hz) band is stronger for attention allocation during real driving conditions (Li, 2010). Hence, we select θ, α, and β bands as the neurophysiologic indexes for characterizing fatigue and awake mental states. For each segment, one-sided PSD estimation to obtain the PSD features at three selected frequency bands, is shown in Figure 3B. Since the frequency ranges of EEG signals in θ, α, β bands are 4–7, 8–13, and 14–30 Hz, respectively, thus, we can get 27 frequency points at each integer frequency to calculate corresponding PSD features. All the frequencies in 61 channels are appended together to form 61 × 27 = 1,647 characterizes (Figure 3C). Specifically, the feature dimension of each subject we finally extracted is 1,400 × 1,647.

## 4. Method

**Figure 4:**
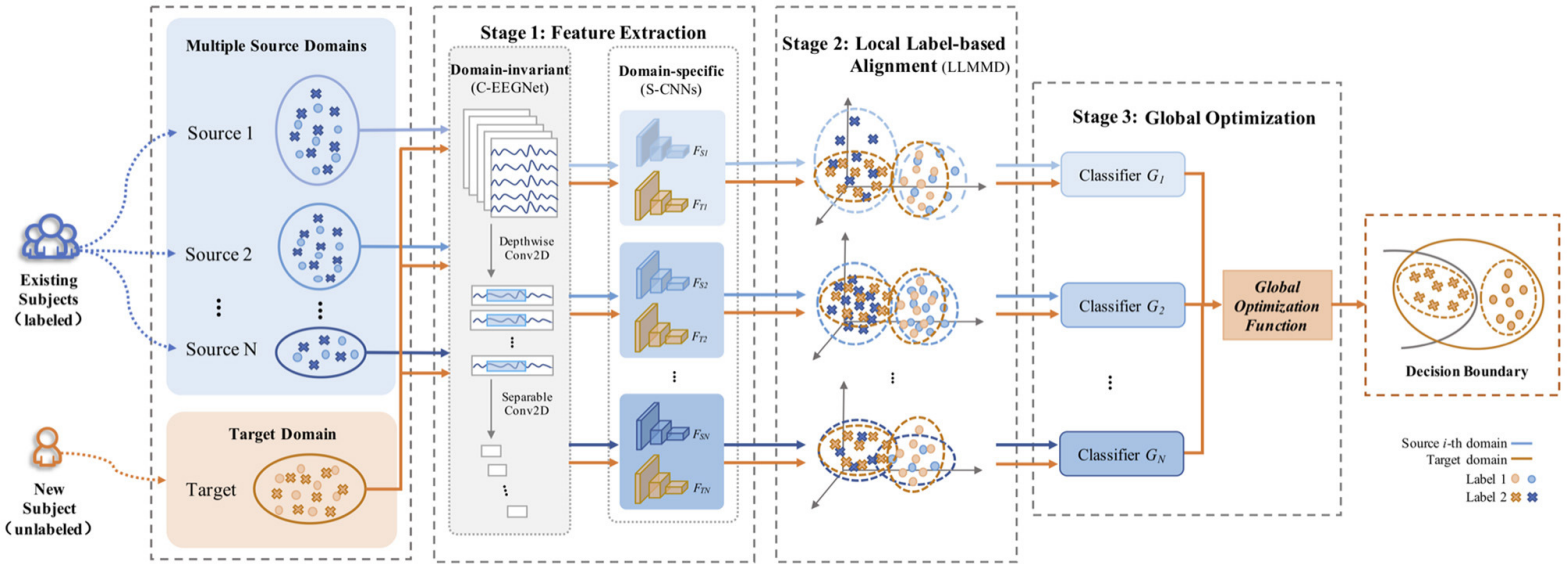
The framework of Label-based Alignment Multi-Source Domain Adaptation (LA-MSDA).

**Figure 5:**
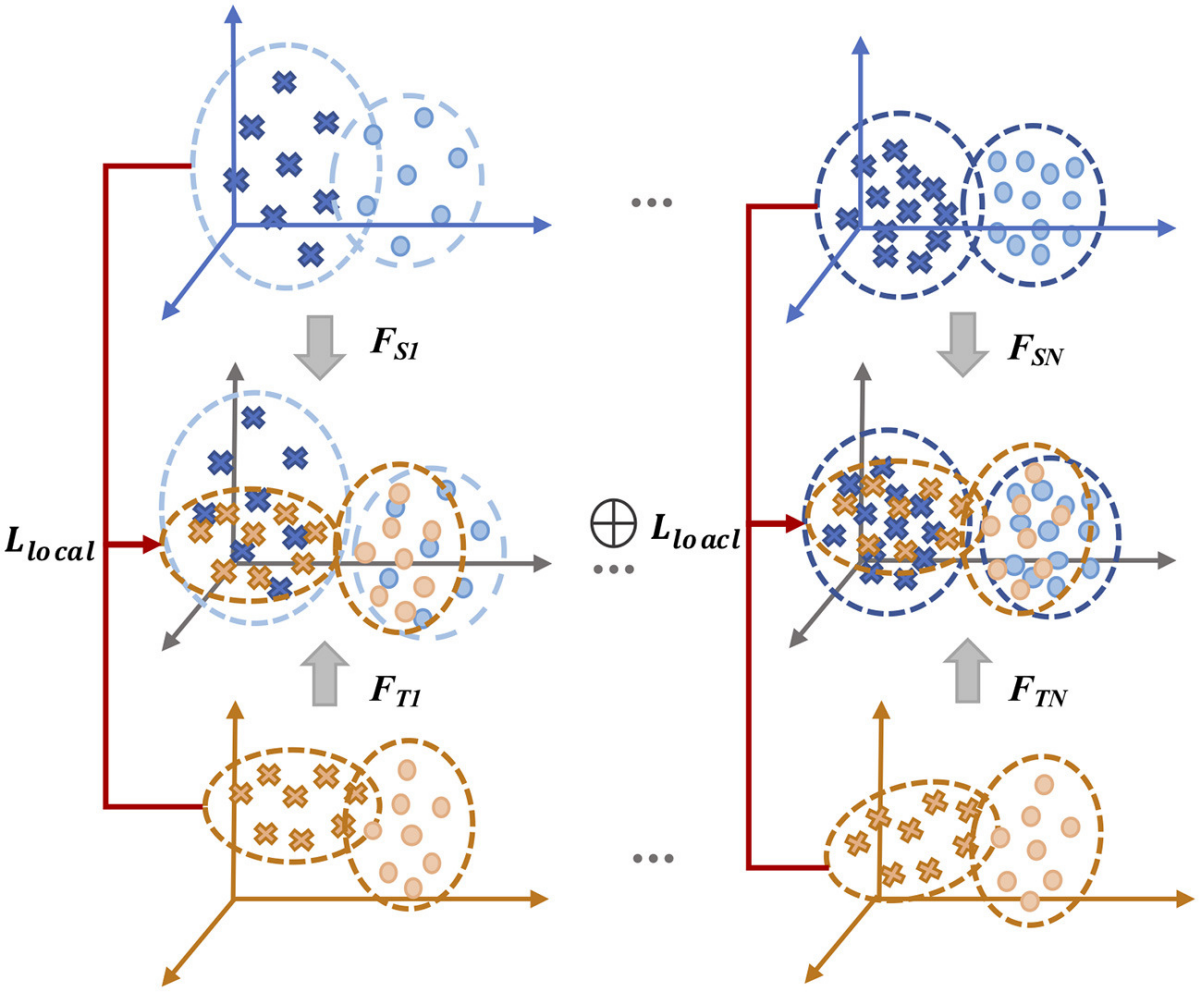
The framework of Local Label-based Maximum Mean Discrepancy (LLMMD).

**Figure 6:**
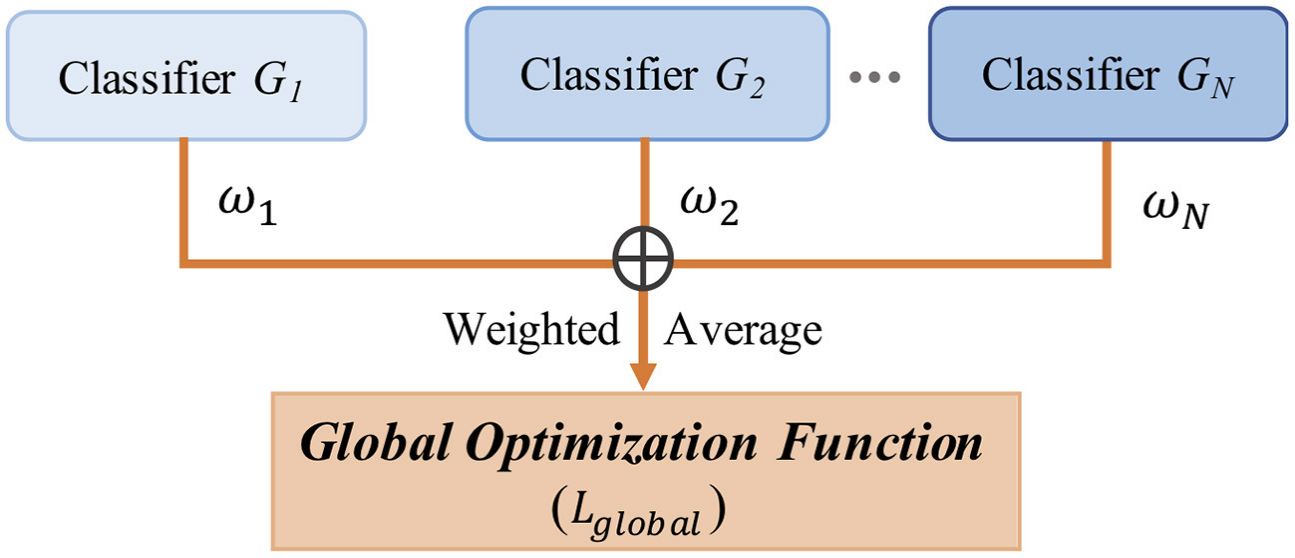
The framework of global optimization.

LA-MSDA is composed of three stages, as illustrated in Figure 4. The first stage is feature extraction, which aims to extract common domain-invariant features from all source and target domains, as well as domain-specific features from each pair of source and target domains. These features are extracted by several networks, including a common EEGNet-based network (C-EEGNet) (Lawhern et al., 2018) and multiple CNN-based subnets (S-CNNs) that do not share the weights. Due to the significant individual differences of EEG across subjects, it is hard to learn specific features for each subject directly (one subject is regarded as one domain in this study). Therefore, we firstly extract common domain-invariant features for all domains by C-EEGNet. Then, the common domain-invariant features are sent to S-CNNs, which map into specific feature spaces to achieve domain-specific features. Technically, the number of S-CNNs is equal to that of source domains. To eliminate the negative impact of significant individual differences among different subjects, we consider the local feature distributions of relevant labels between each pair of the source and target domains. Hence, the second stage introduced the local label-based alignment strategy to align the label-based fine-grained feature distributions in both source and target domains. In the alignment process, adding label-based weight constraints by the Local Label-based Maximum Mean Discrepancy (LLMMD) method (Please refer to Figure 5 for details) can efficiently extract label-based domain-invariant features. For each S-CNNs, we train a domain-specific classifier. Due to inconspicuous features near the decision boundary, the target sample might get a different label predicted by different classifiers. Consequently, in the third stage, the global optimization of all classifiers will be performed (Please refer to Figure 6 for details). It addresses the classifier confusion decision boundary issues by aligning the prediction distributions of the target samples output of each domain-specific classifier. Then, according to the prediction distributions, the similarity weight constraints are set to improve the generalization ability of LA-MSDA in cross-subject EEG analysis. To make the narration clearer, we have the following detailed notations:

- *N*: subjects number, as well as source domains number.
- *U*_*S*_: labeled multi-source dataset, $U_{S}={\left\{\left(X_{n}^{S},Y_{n}^{S}\right)\right\}}_{n=1}^{N}$, where $X_{n}^{S}={\left\{x_{i}^{sn}\right\}}_{i=1}^{\left|X_{sn}\right|}$ indicates samples from the *n*-th source domain, $Y_{n}^{S}={\left\{y_{i}^{sn}\right\}}_{i=1}^{\left|X_{sn}\right|}$ is the corresponding ground-truth labels, |*X*_*sn*_| is the sample number of the *n*-th source domain, and “S/s” represents the source domain.
- *P*: sample distributions of multi-source domains, $P={\left\{P_{n}^{S}\left(x_{i}^{sn}\right)\right\}}_{n=1}^{N}$, where $P_{n}^{S}\left(x_{i}^{sn}\right)$ is the distribution of sample *x*_*i*_ from the *n*-th source domain.
- *U*_*T*_: unlabeled target dataset, $U_{T}={\left\{x_{i}^{t}\right\}}_{i=1}^{\left|U_{T}\right|}$, where $x_{i}^{t}$ is the *i*-th sample of the target domain, total numbers of the target data is |*U*_*T*_|, and “T/t” represents the target domain.
- *Q*: sample distributions of the target domain, $Q=\left\{Q_{T}\left(x_{i}^{t}\right)\right\}$ is the distribution of the *i*-th sample in the target domain.
- *G*: all classifiers of LA-MSDA, $G={\left\{G_{n}\right\}}_{n=1}^{N}$, where *G*_*n*_ is the *n*-th classifier.

### 4.1. Domain-Invariant and -Specific Features Extraction

**Domain-invariant features extraction**. Given a common model C-EEGNet *f(x)*, the potential domain invariant features of all domains are extracted by mapping these features to a common feature space. C-EEGNet consists of *depthwise* and *separable* convolutions, which are not only suitable for a small number of samples but also can produce interpretable features. So C-EEGNet has strong generalization ability and higher performance for EEG analysis. Finally, we obtain domain-invariant features $f\left(x_{i}^{sn}\right)$ and $f\left(x_{i}^{t}\right)$ from the *n*-th source domain and target domain by C-EEGNet.

**Domain-specific features extraction**. After acquiring the domain-invariant features, we further extract the domain-specific features from each pair of source and target domains by *N* S-CNNs. The domain-invariant and -specific features extraction can efficiently learn between-domain invariant features and within-domain specific features, in addition to many other benefits such as minimize the differences across subjects. These unshared S-CNNs map each pair of source and target domain distributions into a specific feature space, which can extract within-domain specific features. $f\left(x_{i}^{sn}\right)$ as the input of S-CNNs *F*_*n*_(·) to receive domain-specific features $F_{n}\left(f\left(x_{i}^{sn}\right)\right)$ (simplified as ${\tilde{x}}_{i}^{sn}$) of the *n*-th source domain, as well as feed $f\left(x_{i}^{t}\right)$ to the *n*-th S-CNNs to get specific features $F_{n}\left(f\left(x_{i}^{tn}\right)\right)$ (simplified as ${\tilde{x}}_{i}^{tn}$) of the target domain.

For each S-CNNs, we train a classifier *G*_*n*_, *n* = {1, 2, ..., *N*}, which is constructed as *G*_*n*_ = *O*_*n*_ ∘ *F*_*n*_ (∘ represents function composition), where *O*_*n*_ outputs the predictions based on the extracted potential domain-specific features ${\tilde{x}}_{i}^{n}$ from the *n*-th S-CNNs. In the supervised learning process, we add a classification loss for each classifier. This loss learns the ideal value of all weights and deviations through labeled samples from multi-source domains and tries to find a way that aims to minimize the loss. Technically, we formulate the supervised loss of multi-source domains as:


(1) $$\begin{array}{l} {ℒ}_{c}=\underset{{\;}_{N\cdot F\cdot f}}{\text{min}}\sum_{n=1}^{N}=\left(\;\frac{1}{\left|X_{sn}\right|}\sum_{i=1}^{\left|X_{sn}\right|}J(G_{n}({\tilde{x}}_{i}^{sn}),y_{i}^{sn}\right) \end{array}$$

where J(·) is the cross-entropy loss function (Shore and Johnson, 1980).

### 4.2. Local Label-Based Alignment

To diminish the discrepancy among each domain, we propose a novel alignment algorithm called LLMMD that is based on the Maximum Mean Discrepancy (MMD) (Tzeng et al., 2014), LLMMD framework is shown in Figure 5. The basic idea of MMD is that if all statistics are the same, then the two distributions are consistent. MMD can measure the distance between two different but related distributions (Yan et al., 2017). MMD has been widely used to construct regular terms to constrain the learned representation during feature learning in domain adaptation so that the features on each pair of domains are as the same possible. Following previous works (Zhu et al., 2019), MMD between the dataset *X*_*S*_ and the dataset *X*_*T*_ is defined as:


(2) $$\begin{array}{l} D_{ℋ}(X_{S},X_{T})=\underset{\|\phi {\|}_{ℋ\leq 1}}{\sup}\|E_{p}[\phi (X_{S})]-E_{q}[\phi (X_{T})]{\|}_{ℋ}^{2}\\ \;={\left\|\frac{1}{n}\sum_{i=1}^{n}\phi \left(x_{i}\right)-\frac{1}{m}\sum_{j=1}^{m}\phi \left(x_{j}\right)\right\|}_{ℋ}^{2}\\ \;=\|\frac{1}{n^{2}}\sum_{i=1}^{n}\sum_{j=1}^{n}k\left(x_{i},x_{j}\right)+\frac{1}{m^{2}}\sum_{i=1}^{m}\sum_{j=1}^{m}k\left(x_{i},x_{j}\right)\\ {\;-\frac{2}{nm}\sum_{j=1}^{n}\sum_{j=1}^{m}k\left(x_{i},x_{j}\right)\|}_{ℋ} \end{array}$$

where *sup* is to find the upper found, the set of samples $X_{S}={\left\{x_{i}^{s}\right\}}_{i=1}^{n}$ and $X_{T}={\left\{x_{j}^{t}\right\}}_{j=1}^{m}$ from distributions *p* and *q* respectively, and ϕ(·) represents the feature mapping function that maps the distribution of the domain-specific features to the reproducing kernel hilbert space (RKHS) H. Each kernel function *k* corresponds to n RKHS. We use Gaussian Kernels Function $k\left(x_{i},x_{j}\right)=e^{\frac{-||x_{i}-x_{j}|{|}^{2}}{2\sigma^{2}}}$ as the kernel function (σ: Gaussian filter width), which can map an infinite-dimensional space.

Many previous UDA works mainly focus on global features alignment directly, which are hard to perform well due to the significant individual differences of EEG. To enhance generalization ability, we take the features of local label-based distributions into consideration among each pair of domains. Theoretically, LLMMD explores the local label-based fine-grained structure information for all domains and extract label-based domain-invariant features by aligning the distributions of that information. In addition, local label-based alignment matches the distribution not only between source domains but also among each pair of both source and target domains. Overall, LLMMD can improve the capability of multi-source domain adaptation to overcome the limitations of significant individual differences between subjects.

For cross-subject analysis, the categories of the label in EEG of each subject are the same, but there may be a problem of label category weight deviation. Additionally, another challenge is that the samples to be predicted in the target domain are unlabeled. To overcome these issues, we take into account the label categories of different samples for aligning the domain-specific feature distributions in each domain, which can efficiently extract label-based domain-invariant features. With the requirement of local label-based alignment, we assume that the weight φ^*c*^ is the probability that the samples belong to each of *c* label categories, then LLMMD can be denoted as:


(3) $$\begin{array}{l} {ℒ}_{local}=ℒ(D_{ℋ}(X,Y),\varphi^{c})\\ \;=\frac{1}{N}\sum_{n=1}^{N}[\frac{1}{C}\sum_{c=1}^{C}\left|\right|\frac{1}{{\left|x_{n}^{s}\right|}^{2}}\sum_{i=1}^{\left|X_{n}^{s}\right|}\sum_{j=1}^{\left|X_{n}^{s}\right|}\varphi_{i}^{cs}\varphi_{j}^{cs}k\left({\tilde{x}}_{i}^{sn},{\tilde{x}}_{j}^{sn}\right)\\ \;-\frac{2}{\left|x_{n}^{s}\right|\left|x_{t}\right|}\sum_{i=1}^{\left|X_{n}^{s}\right|}\sum_{j=1}^{\left|X_{t}\right|}\varphi_{i}^{cs}\varphi_{j}^{ct}k\left({\tilde{x}}_{i}^{sn},{\tilde{x}}_{j}^{t}\right)\\ \;+\frac{1}{{\left|x_{t}\right|}^{2}}\sum_{i=1}^{\left|X_{t}\right|}\sum_{j=1}^{\left|X_{t}\right|}\varphi_{i}^{ct}\varphi_{j}^{ct}k\left({\tilde{x}}_{i}^{t},{\tilde{x}}_{j}^{t}\right)\|] \end{array}$$

where φ^*cn*^ and φ^*ct*^ represent the local label categories weight of ${\tilde{x}}_{i}^{sn}$ and ${\tilde{x}}_{i}^{t}$ assigned to the label category *c* in each domain of *U*_*s*_ and *U*_*t*_, respectively. Based on the label category prior distributions, the set φ^*cs*^ of multi-source domains is defined as:


(4) $$\begin{array}{l} \varphi^{cs}:=\overset{N}{\underset{n=1}{⋃}}\varphi^{cn},\varphi^{cn}=\overset{\left|X_{n}^{s}\right|}{\underset{i=1}{⋃}}\frac{y_{i}^{cn}}{\Sigma_{y_{j}\in Y_{n}}y_{j}^{cn}},\underset{\varphi_{i}\in \varphi^{cn}}{\sum }\varphi_{i}^{cn}=1 \end{array}$$

where $y_{i}^{cn}$ is the true label of the sample *y*_*i*_ in the *n*-th source domain belonging to the *c*-th label category, *i* and *j*, respectively, denote the sample index in the dataset in the *c*-th label category. However, in the target domain, we cannot get the label-based structure information directly due to a lack of labels. The similar feature distributions between different domains mean that the classifier *G*_*n*_ trained on each source domain can predict most of the probability distribution of target samples correctly. Therefore, for unlabeled target subject *U*_*t*_, using the output of *n*-th classifier *G*_*n*_ as the probability distribution ${ŷ}_{i}^{cg}$ of the target sample ${\tilde{x}}_{i}^{ct}$ pertain to the local label category *c*. In this setting, the target sample ${\tilde{x}}_{i}^{ct}$ is weighted as:


(5) $$\begin{array}{l} \varphi_{i}^{ct}:=\overset{N}{\underset{g=1}{⋃}}\varphi_{i}^{cg}=\overset{N}{\underset{g=1}{⋃}}\frac{{\hat{y}}_{i}^{cg}}{\sum_{\left(x_{j},{\hat{y}}_{j}\right)\in U_{t}}{\hat{y}}_{j}^{cg}},\sum_{\varphi_{i}\in \varphi^{cg}}\varphi_{i}^{cg}=1\; \end{array}$$

### 4.3. Global Optimization

From another perspective, we further consider the global distribution discrepancy (Figure 6). For the target samples near the decision boundary, there is a high possibility of being misclassified by the classifiers trained on different source domains, and the prediction distribution for these target samples will be ambiguous from different classifiers. Empirically, the same target samples should obtain the consistent prediction distribution predicted by different classifiers. Hence, to solve the above problem, we align the prediction distributions of target samples output from each classifier, which can efficiently minimize the discrepancy among different classifiers. For EEG data with high non-linearity, the inconspicuous features near the decision boundary can make correct decisions by conducting that of aligning. Formally, we utilize the representation output from different classifiers to calculate the discrepancy loss:


(6) $$\begin{array}{l} H_{g}=\frac{2}{N\left(N-1\right)}\overset{N-1}{\underset{n=1}{\sum }}\overset{N}{\underset{m=n+1}{\sum }}\left[\frac{1}{\left|X_{t}\right|}\overset{\left|X_{t}\right|}{\underset{i=1}{\sum }}|G_{n}\left({\tilde{x}}_{i}^{t}\right)-G_{m}\left({\tilde{x}}_{i}^{t}\right)|\right] \end{array}$$

Due to the significant individual differences of EEG, if the average prediction results of all source domain classifiers are direct as the objective function, it will be difficult to reach the optimal state and may fall into a local optimal state. Therefore, we introduce a global objective function optimization strategy to improve the generalization ability of the proposed model in cross-subject EEG analysis. Theoretically, we consider the similarity between subjects, setting similarity weight constraints according to the prediction probability distribution results of each classifier. Based on the weighted average strategy (Polikar, 2012; Wang et al., 2014), the smaller the discrepancy between two classifiers, the higher the weight. Furthermore, the global optimization strategy can also efficiently eliminate the negative impact of significant individual differences. Therefore, the whole method integrates the probability distribution from *N* classifiers by the weighted mechanism. In global optimization, the global classifiers discrepancy loss can be calculated based on the weight ω in the following equation:


(7) $$\begin{array}{l} \omega_{m}^{N}=\overset{N}{\underset{n=1}{⋃}}\omega_{m}^{n}=\overset{N}{\underset{n=1}{⋃}}\frac{G_{n}\left({\tilde{x}}_{i}^{t}\right)-G_{m}\left({\tilde{x}}_{i}^{t}\right)}{\sum_{j\in N,j\neq n}\left(G_{n}\left({\tilde{x}}_{i}^{t}\right)-G_{j}\left({\tilde{x}}_{i}^{t}\right)\right)} \end{array}$$

where $\omega_{m}^{n}$ represents the discrepancy loss weight between the *n*-th classifier and the *m*-th classifier. Finally, the ensemble of all classifiers with the constraint of weight $\omega_{m}^{n}$ that can reformulate (Equation 6) is as follows:


(8) $$\begin{array}{l} {ℒ}_{global}=L(H_{g},\omega_{m}^{n})\\ \;=\frac{2}{N(N-1)}\sum_{n=1}^{N-1}\sum_{m=n+1}^{N}\left(1-\omega_{m}^{n}\right)[\frac{1}{\left|X_{t}\right|}\sum_{i=1}^{\left|X_{t}\right|}|G_{n}\left({\tilde{x}}_{i}^{t}\right)\\ \;-G_{m}\left({\tilde{x}}_{i}^{t}\right)|] \end{array}$$

### 4.4. Label-Based Alignment Multiple Sources Domain Adaptation

Label-based Alignment Multi-source Domain Adaptation model is a novel UDA model for more effective adaptation. The goal of UDA is to learn domain-invariant features, so LA-MSDA first extracts domain-invariant and -specific features by several networks to achieve better performance in cross-subject fatigue mental state analysis. Specifically, to eliminate the negative impact of high non-linearity and significant individual differences, we introduce a local label-based alignment loss $L_{local}$ to extract label-based domain-invariant features by aligning the label-based fine-grained feature distributions of each domain, and a global classifiers discrepancy loss $L_{global}$ to align the outputs of the domain-specific classifiers and integrate all classifiers by adding the similarity weight constraints, which address the issue of classifier confusion decision boundary and improve the generalization ability of LA-MSDA. Therefore, we propose to train LA-MSDA by optimizing the following objective function:


(9) $$\begin{array}{l} {ℒ}_{total}={ℒ}_{c}+\mu {ℒ}_{local}+\gamma {ℒ}_{global} \end{array}$$

where the hyper-parameter μ and γ set a relative trade-off, respectively.

## 5. Experiments

In this section, we evaluate the LA-MSDA method and compared its performance with state-of-the-art DL and TL. The experiments are conducted on an NVIDIA GeForce RTX 3080 graphics processor with 10 GB of memory, and the algorithms have been verified with Python 3.7 tools under the environment of windows10.

### 5.1. Setup

**Dataset**. The dataset includes EEG recording of 15 subjects by the industry and neural science laboratory in University of Rome Sapienza, the details are shown in section 3.

**LA-MSDA architecture**. EEGNet-based networks are used as the backbone of LA-MSDA, and we fine-tune all layers of EEGNet and train the classifier with a learning rate of 0.001 and the batch size of 64. The input data of LA-MSDA have been pre-processed by PSD (refer section 3.2).

**Baselines**. In our experiments, there are three categories of baselines: (1) *traditional ML* methods, such as Support Vector Machines (**SVM**) (Chang and Lin, 2011); (2) *single-source UDA* methods, including Domain-adversarial Neural Network (**DANN**) (Ganin et al., 2016) and Deep Subdomain Adaptation Network (**DSAN**) (Zhu et al., 2020); (3) *multi-source UDA* methods, including Multiple Feature Spaces Adaptation Network (**MFSAN**) with **ResNet-50** (Zhu et al., 2019). For each model, we perform 15 times of experiments to evaluate the performance, and the input of each model is the same training set and testing set. SVM is the most typical traditional ML method that can be used to highlight the performance of TL. To further demonstrate the powerful performance of multi-source domain adaptation in the UDA filed, the common single-source UDA methods DANN and DSAN are introduced as comparative experiments. For the existing multi-source UDA methods, the MFSAN method use ResNet-50 to train multiple classifiers, and aligning domain-specific distribution and classifier for multi-source domains classification. However, this model is not efficient to train because it takes a long time and the local- and global-based information are not taken into account. To improve the effectiveness of training, LA-MSDA utilizes EEGNet as the main network. In addition, we consider the label-based fine-grained structure information and global optimization to improve the generalization ability of LA-MSDA in cross-subject EEG analysis. Our code will be available at https://github.com/PyTorchTL/LA_MSDA.git.

To further validate the effectiveness of different modules, we also evaluate several variants of LA-MSDA: (1) **Ours(E)**, only considering stage 1 of LA-MSDA with EEGNet-based network; (2) **Ours(E+L)**, considering both stage 1 and stage 2 of LA-MSDA; (3) **Ours**, considering the whole LA-MSDA framework through all three stages.

### 5.2. LA-MSDA Evaluation

#### 5.2.1. Evaluation of The Number of The Source Domains

**Figure 7:**
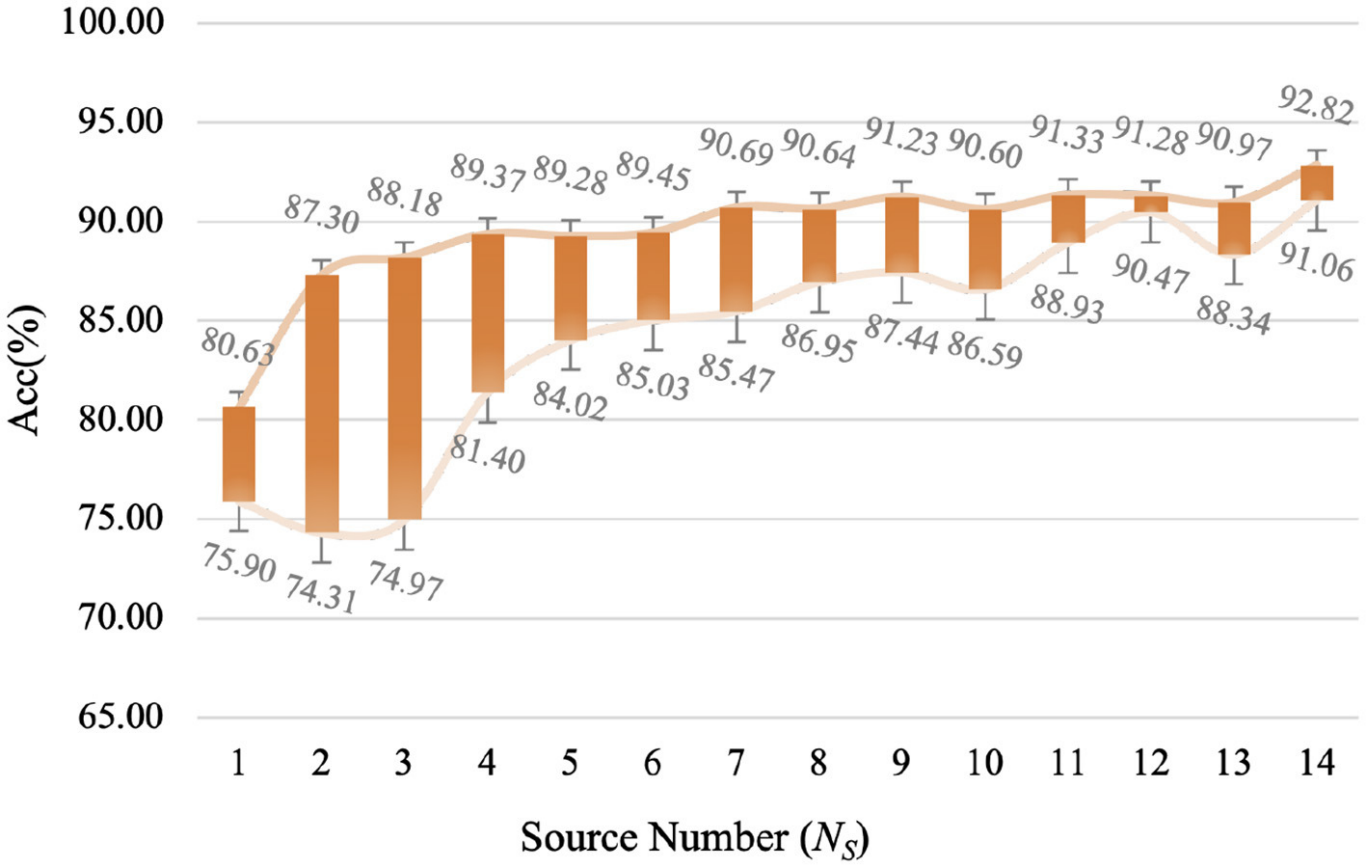
The highest and lowest accuracy obtained under different source numbers of LA-MSDA. The “Acc” value represents the average accuracy for all subjects under different source numbers.

For multiple sources UDA, the parameter of source number *N*_*S*_ is also an important factor. In this study, a subject is regarded as a source domain, which means that 15 subjects correspond to 15 source domains. Additionally, the source number is also equal to that of classifiers, in other words, selecting more sources will train more classifiers. Therefore, we analyze the impact of different source numbers on the performance. Due to various restrictions, we cannot analyze all situations (all combinations of source domains dataset), so in this study, we select the best situation (i.e., selecting the most similar *N*_*S*_ subjects as the source domains dataset) and the worst situation as the floating interval of model accuracy, as shown in Figure 7. We can find that the fluctuation of accuracy tends to be stable with the increase of source number. When the number of source domains is 12, LA-MSDA is the most stable, indicating that the model can most efficiently eliminate the influence of individual differences for cross-subject EEG. In addiction, LA-MSDA achieves the highest accuracy and better stability when *N*_*S*_ is 14. Thus, we set *N*_*S*_=14 in the following experiments.

#### 5.2.2. Auxiliary Training Data Amount

**Figure 8:**
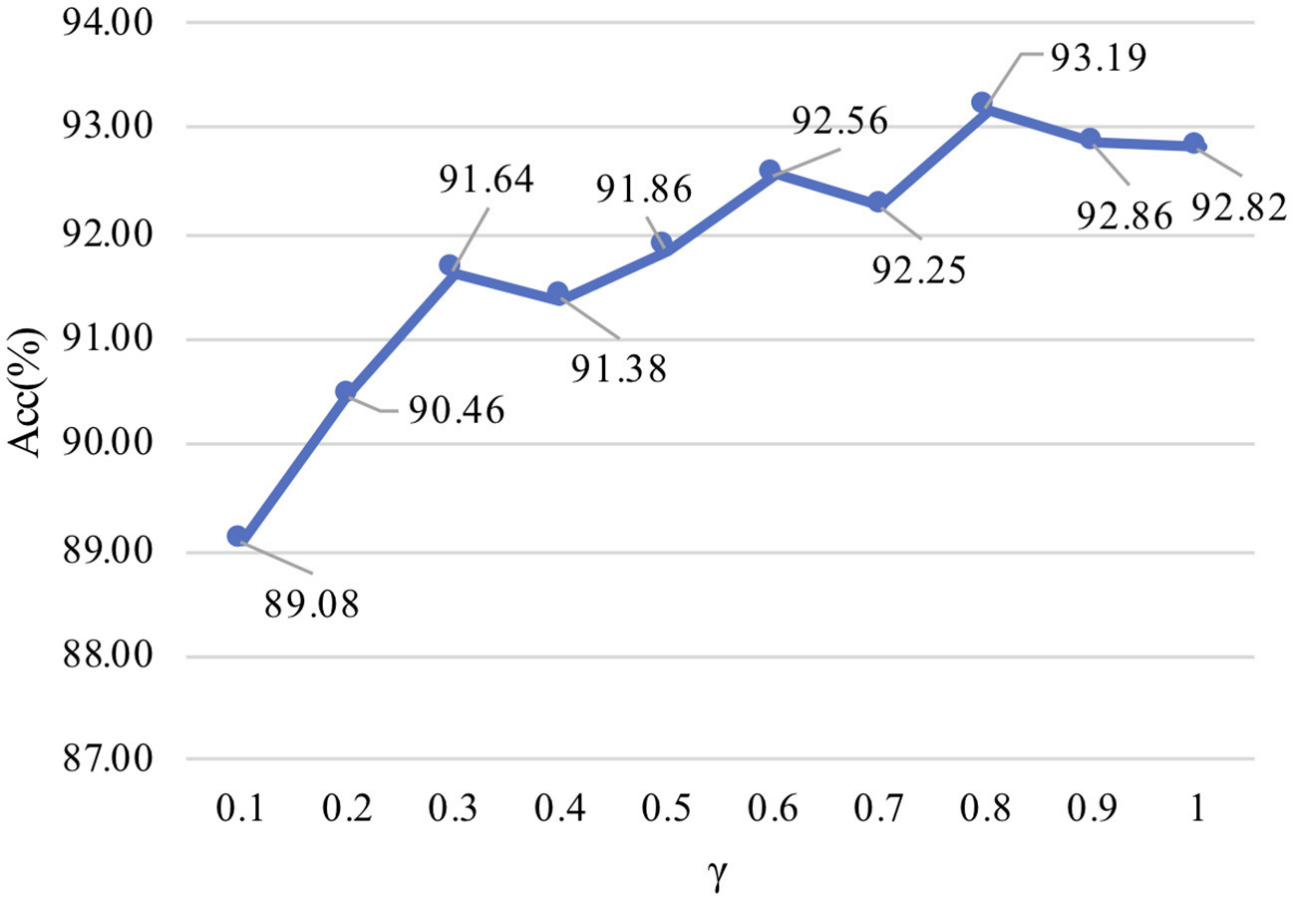
Auxiliary training data amount. The “Acc” value represents the average accuracy for all subjects under different ratios.

For unlabeled target samples, we set the parameter of γ, that is, the auxiliary training data from unlabeled target samples are randomly picked out, where the auxiliary training data (unlabeled) are used to assist training classifiers with the labeled source domains. The remaining samples of the target are used for testing. The influence of different auxiliary training data amount is shown in Figure 8. With the increase of auxiliary training data rate in target, the average accuracy also gradually improved. When γ is 0.8, the corresponding average accuracy for the best performance is 93.19%. The results show that auxiliary training data can be used to assist the training classifiers to of source domains obtain better performance.

For the parameter of γ, the best performance of average accuracy was obtained with γ = 0.8, and the accuracy is slightly reduced when γ is 1. However, when γ is 0.8, the model converges slowly, which takes more time to be stable. Considering the above factors, we finally set γ to 1 to balance the model convergence speed and accuracy.

#### 5.2.3. Individual Performance

To show the results more intuitively for LA-MSDA, we compare its performance with the above-mentioned baseline models (refer section 5.1). Table 2 summarizes the results on the 15 subjects, for each experiment, one subject (unlabeled) as the target testing samples and the others (labeled) as the sources training set. The baseline of each model is the average accuracy of all subjects tested by this model, and the dataset of each model is consistent. Notably, the single-source network means that all source subjects together form just one source domain, and the multi-source domains assume that each subject regarded as a source domain, respectively, then there will be 14 source domains of 14 subjects for training.

**Table 2:** Classification accuracy performance of individuals (%).

**Subject** **ID**	**ML**	**Single-Source**		**Multi-Source**			
	**SVM**	**DANN**	**DSAN**	**MFSAN**	**Ours(E)**	**Ours(E+L)**	**Ours**
N1	64.21	72.64	72.64	89.86	93.25	93.57	93.86
N2	80.71	86.29	80.36	88.36	95.75	92.86	96.21
N3	60.29	91.14	94.64	98.57	99.04	98.57	99.29
N4	57.50	90.21	85.57	94.36	95.68	94.36	97.00
N5	56.14	87.71	80.00	83.29	90.04	92.14	92.21
N6	55.00	92.57	88.00	98.43	99.07	96.43	99.57
N7	63.07	67.29	69.07	61.93	83.32	89.29	89.71
N8	63.14	77.57	79.00	79.07	76.61	87.86	81.57
N9	73.57	87.57	93.29	87.00	90.29	90.71	90.93
N10	76.43	91.00	81.07	94.21	95.39	96.43	97.29
N11	52.07	60.86	60.93	68.36	86.00	80.71	86.36
N12	63.29	85.93	81.57	76.07	86.5	90.00	92.00
N13	34.57	66.93	67.71	68.86	62.14	80.00	87.21
N14	49.29	65.00	58.07	74.64	85.00	86.43	92.57
N15	75.50	56.64	81.43	90.64	93.86	94.29	96.5
Avg^*^	61.65	78.62	78.22	83.58	**88.80**	**90.91**	**92.82**

* *Avg, average value. The bold values: highlight the results of our method*.

Compared with various methods, the results show that LA-MSDA achieves the highest average accuracy of 92.82%, where that of each individual is also the highest. For multi-source UDA works, LA-MSDA is higher than MFSAN by 9.24%. The mean accuracy rises more than 14.6% when compared to single-source UDA methods of DANN and DSAN. The sharpest rise is 31.17%, which is the result of comparison between LA-MSDA and SVM.

Furthermore, we add the ablation experiments to further validate the effectiveness of LA-MSDA. The experimental results show that each module we proposed has improved the model performance. EEGNet-based network of Ours(E) is higher than MFSAN based on ResNet-50 by 5.22%, and they also have a significant reduction in training time (refer to **Figure 11**). Ours(E+L) by adding the LLMMD module (stage 2), the model performance is improved by 2.11% based on Ours(E). Finally, the whole model of Ours (LA-MSDA) considering all three stages reached the highest accuracy rate of 92.82%.

#### 5.2.4. Confidence Evaluation

For multi-source UDA methods, based on the confusion matrix, we select Accuracy, Precision, F1Score, and Recall as metrics to further evaluate the individual performance between MFSAN and Ours (LA-MSDA), as shown in Table 3. From the aspects of these four metrics, LA-MSDA outperforms the compared multi-source domain method MFSAN not only in the average value, but also in the evaluation value of each subject. Overall, the results indicate the effectiveness of LA-MSDA for cross-subject EEG fatigue mental state evaluation.

**Table 3:** The performance of Accuracy, Precision, F1Score, and Recall for multi-source models of each subject (%).

**Subject** **ID**	**Accuracy**		**Recall**		**Precision**		**F1Score**	
	**MFSAN**	**Ours**	**MFSAN**	**Ours**	**MFSAN**	**Ours**	**MFSAN**	**Ours**
N1	89.86	93.86	88.46	92.49	88.59	92.89	88.71	93.29
N2	88.36	96.21	90.01	97.22	88.11	96.02	86.29	94.86
N3	98.57	99.29	98.13	99.00	97.92	99.07	97.71	99.14
N4	94.36	97.00	93.84	96.44	93.70	96.58	93.57	96.71
N5	83.29	92.21	80.84	93.32	81.42	91.56	82.00	89.86
N6	98.43	99.57	98.27	98.59	97.85	99.15	97.43	99.71
N7	61.93	89.71	58.46	89.97	57.35	89.19	56.29	88.43
N8	79.07	81.57	76.18	80.59	76.23	81.22	76.28	81.86
N9	87.00	90.93	84.94	92.62	85.18	90.18	85.43	87.86
N10	94.21	97.29	93.31	95.26	93.51	96.47	93.71	97.71
N11	68.36	86.36	68.34	85.07	68.10	85.67	67.86	86.29
N12	76.07	92.00	74.72	93.85	74.93	91.59	75.14	89.43
N13	68.86	87.21	67.69	85.28	68.55	86.48	69.43	87.71
N14	74.64	92.57	72.30	94.61	73.42	92.40	74.57	90.29
N15	90.64	96.50	91.04	95.83	90.52	95.48	90.00	95.14
Avg^*^	83.58	**92.82**	82.44	**92.68**	82.36	**92.26**	82.29	**91.89**

* *Avg, average value. The bold values: highlight the results of our method*.

**Figure 9:**
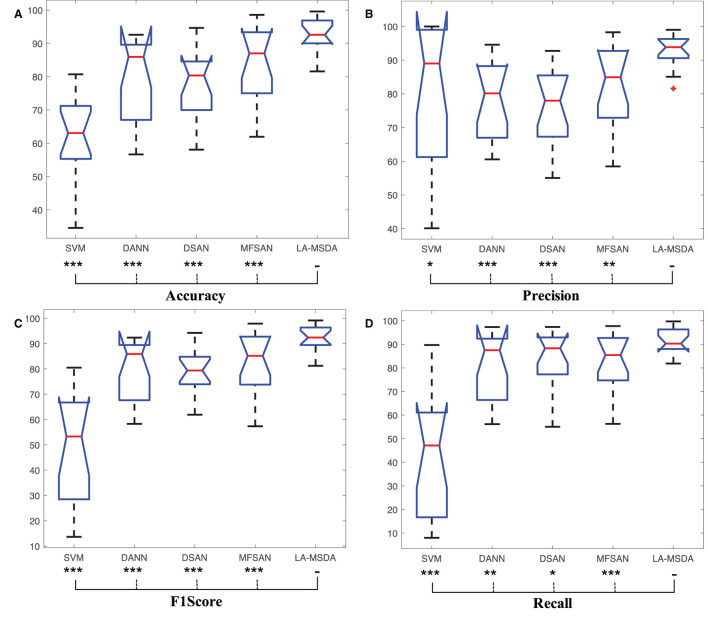
Confidence evaluation. Box plots: four related metrics based on confusion matrix to compare LA-MSDA with Support Vector Machines (SVM), Domain-adversarial Neural Network (DANN), Deep Subdomain Adaptation Network (DSAN), and Multiple Feature Spaces Adaptation Network (MFSAN). **(A)** Represents the confusion matrix of Accuracy. **(B)** Represents the confusion matrix of Precision. **(C)** Represents the confusion matrix of F1Score. **(D)** Represents the confusion matrix of Recall. *p-value between different models. (*p<0.05; **p<0.01; ***p<0.001).

Furthermore, the four metrics of LA-MSDA and the comparison methods are analyzed by Wilcoxon Sign-Rank Test, and the performance of significant differences is shown in Figure 9. LA-MSDA is superior to all comparison methods (*p* < 0.05 for all metrics). The *p*-values also show that there are significant differences between these comparison methods.

#### 5.2.5. Convergence Evaluation

**Figure 10:**
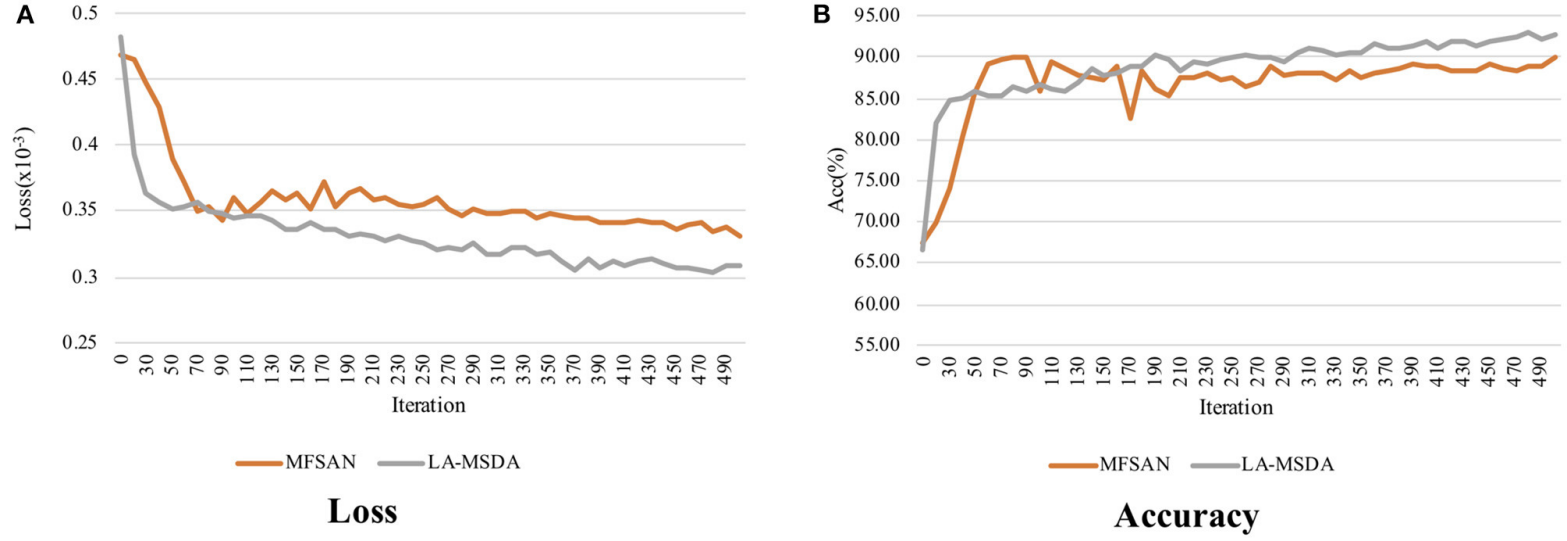
Convergence of MFSAN and LA-MSDA on target domain data subject N1. **(A)** The total loss performance on subject N1 during the increase of iteration of MFSAN and LA-MSDA, which shows LA-MSDA can converge faster; **(B)** Compared with MFSAN, LA-MSDA can achieve higher accuracy on subject N1.

We further analyze the convergence of MFSAN and LA-MSDA, the loss and accuracy are shown in Figure 10. Taking the subject N1 as an example, and setting the number of iteration to 500, the results in Figure 10A indicate that the total loss of LA-MSDA achieves faster convergence under the same number of iterations. From Figure 10B, with the increase of the iteration numbers, the corresponding accuracy maintains steady growth and is higher than MFSAN.

**Figure 11:**
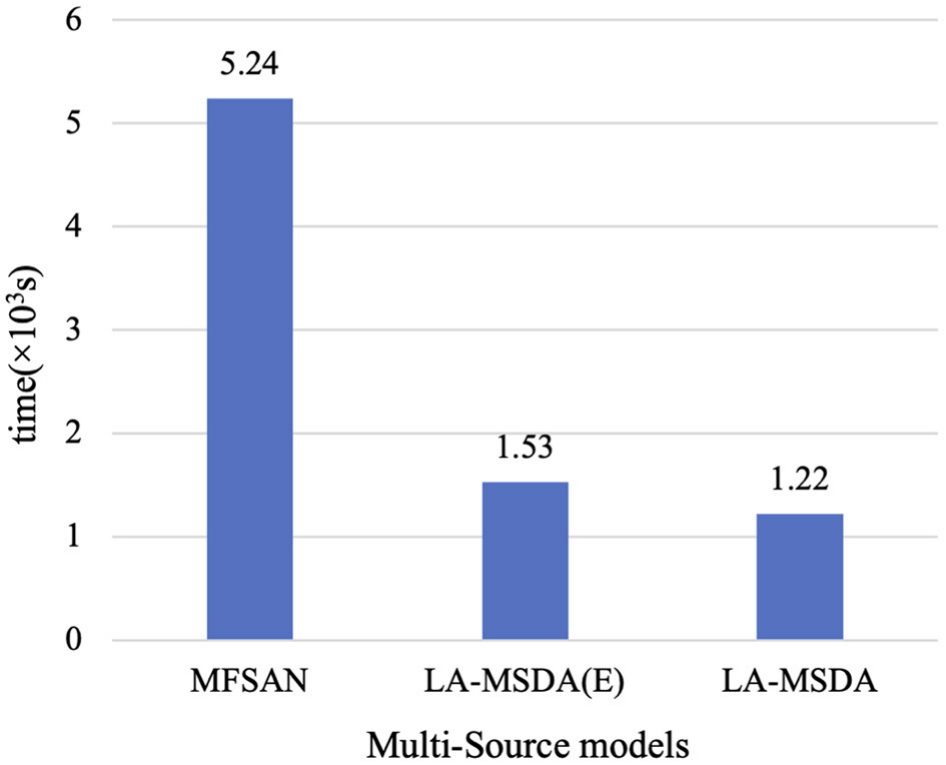
Time of convergence compared with MFSAN, LA-MSDA(E), and LA-MSDA. LA-MSDA(E): only considering stage 1 of LA-MSDA with EEGNet-based network.

In addition, the time of convergence is calculated, as shown in Figure 11. The convergence time means the time for the model to train classifiers until convergence. Since LA-MSDA is an improved model based on a multi-source domain, we compare its convergence time with that of the existing multi-source domain models. LA-MSDA requires much less time than ResNet-based MFSAN, and slightly less than LA-MSDA(E). It can be concluded that an EEGNet-based network can greatly reduce the model convergence time, and our proposed algorithms can further accelerate the model convergence speed. The results verify that LA-MSDA can achieve high-efficiency and high-precision fatigue mental state evaluation.

## 6. Discussion

### 6.1. Parameter Sensitivity

For LA-MSDA, we investigate the sensitivity of different parameters, including source number *N*_*S*_ and auxiliary training data ratio λ. To evaluate the sensitivity of source number, we record the performance of LA-MSDA under different source numbers. We calculate the classification accuracy interval of LA-MSDA based on the similarity between the source and target domain. That is, in Figure 7, the interval value of the largest and the least is calculated by the top Ns source domains most similar to the target domain and last Ns with the biggest difference, respectively. The interval better reflects the impact of the selection of source domain samples on model performance. For the source number less than 14, whether the selection of source domains is random or most similar, the accuracy fluctuates within the interval corresponding to the source number. Due to the significant individual differences, the performance of the model will decline when the source number is decreasing. However, as the source number increases, the model training time will also increase. Overall, the performance of LA-MSDA tends to be efficient and stable when the source number reaches 6.

To improve the model performance, we can choose to increase the auxiliary training data rate in the target domain to assist the source data training classifiers. In Figure 8, the results show the relationship between λ and accuracy, and what we find is that the auxiliary training data from the target domain can assist LA-MSDA to achieve better performance. With the increase of auxiliary training data amount, the optimal performance of LA-MSDA can be achieved when γ is 0.8, and then the performance may decrease slightly when γ increases to 1. That is due to the significant differences in cross-subject, a larger amount of data does not imply absolute advantage, and may cause a certain degree of negative transfer effect. When the number of unlabeled samples participating in the auxiliary training decreases, the overall performance of our proposed model declines because the label distribution feature between the samples in the source and target domain could not be completely obtained. Therefore, we use all unlabeled samples in the target domain for auxiliary training, and the experimental results also show that our proposed model could also achieve better overall performance in this case.

### 6.2. Compare Individual Performance With Existing Methods

In recent years, various research studies have emerged for evaluating EEG-based mental states. In this study, we choose some typical methods to perform a comparison with LA-MSDA, including SVM, DANN, DSAN, and MFSAN. However, due to the high non linearity and significant individual differences of EEG, their performance is not well for cross-subject. LA-MSDA eliminates the negative impact of that characteristics by achieving local label-based alignment and global optimization for cross-subject EEG. As can be seen from Tables 2, 3, LA-MSDA reaches the highest value, whether it is the average accuracy of all subjects or the accuracy of each subject, and the accuracy fluctuates little among subjects.

The results of multi-source domains are better than single-source domains, which demonstrates that extracting domain-specific features can efficiently eliminate the negative impact of significant individual differences of EEG. Ours(E) outperforms MFSAN results indicate that the EEGNet-based network can not only extract effective features of cross-subject EEG data but also can greatly reduce the training time of the model. The performance of Ours(E+L) is improved based on Ours(E), which demonstrates that the strategy of local label-based alignment is helpful on cross-subject EEG fatigue mental state evaluation. By aligning the label-based fine-grained feature distributions, we can efficiently extract label-based domain-invariant features, thereby eliminating the impact of significant individual differences in EEG. Finally, we introduce a global optimization strategy, and the results show that LA-MSDA is better than all comparison methods. This strategy addresses the issue of inconspicuous features decision boundary and improves the generalization ability of LA-MSDA. In general, our LA-MSDA model can achieve better performance in cross-subject EEG fatigue mental state evaluation.

### 6.3. Model Convergence

We testify that the convergence of LA-MSDA outperforms MFSAN. LA-MSDA converges faster than MFSAN in the same period. Also, the total loss of LA-MSDA is lower, which is the sum of $L_{c}$, $L_{local}$, and $L_{global}$. From the results of convergence, we can find that these two models can almost converge after 300 iterations. Overall, LA-MSDA minimizes the discrepancy between each domain by aligning the local label-based feature distributions and achieving global optimization to get smaller losses and higher accuracy. Meanwhile, to evaluate the efficiency of LA-MSDA, we compare the convergence time between LA-MSDA and MFSAN. MFSAN is a ResNet-based classification method for multiple sources. To indicate the efficiency of the EEGNet-based network for EEG processing, we change the deep ResNet-50 to the shallow EEGNet by fine-tuning all convolution layers and pooling layers. The comparison results show that MFSAN with ResNet-50 takes about four times longer than LA-MSDA with EEGNet tends to convergence, which indicates that the EEGNet-based network plays a leading role in improving the efficiency of LA-MSDA for EEG analysis. By introducing our optimization strategy (stage 2 and stage 3) based on LA-MSDA(E), it can be found from the comparison results that LA-MSDA can still further improve the training efficiency of LA-MSDA(E). Notably, LA-MSDA takes the least time to achieve training and testing with high efficiency.

## 7. Conclusion

In this study, we propose a novel method LA-MSDA to evaluate EEG-based fatigue mental state for the cross-subject, which efficiently eliminates the negative impact of high non-linearity and significant individual differences of EEG. LA-MSDA mainly introduces two optimization strategies, including local label-based alignment and global optimization. For details, the strategy of local label-based alignment by extracting label-based domain-invariant features to eliminate the impact of significant individual differences of EEG. Additionally, the global optimization strategy is introduced to address the inconspicuous features decision issues and improve the generalization ability of LA-MSDA, which can be achieved by aligning the prediction distributions of each classifier and adding the similarity weight constraints. Finally, the experimental results show the superiority of the proposed method.

## Data Availability Statement

The raw data supporting the conclusions of this article will be made available by the authors, without undue reservation.

## Ethics Statement

The study was conducted according to the guidelines of the Declaration of Helsinki, and approved by the Ethics Committee of the Department of Physiology and Pharmacology of Sapienza University of Rome (Roma, 21/4/2016). The patients/participants provided their written informed consent to participate in this study.

## Author Contributions

GD provided the outline of the manuscript. YZ and HZ contributed to search the literatures, study design, and write the manuscript. GB, PA, GDF, FB, and HZ provided the data and resources. JZ, YZ, ZZ, and XL designed the experiments, assembled the setup, contributed to the analysis of the data, and organized experiment results. GD, HZ, and YZ supervised the study and completed the final editing. All authors discussed and approved the submitted manuscript.

## Funding

This study was partly supported by the National Key R&D Program of China with grant no. 2017YFE0118200, NSFC with grant No. 62076083, and Fundamental Research Funds for the Provincial Universities of Zhejiang with grant no. GK209907299001-008.

## Conflict of Interest

The authors declare that the research was conducted in the absence of any commercial or financial relationships that could be construed as a potential conflict of interest.

## Publisher's Note

All claims expressed in this article are solely those of the authors and do not necessarily represent those of their affiliated organizations, or those of the publisher, the editors and the reviewers. Any product that may be evaluated in this article, or claim that may be made by its manufacturer, is not guaranteed or endorsed by the publisher.

## References

[B1] Bhattacharyya S. Sengupta A. Chakraborti T. Konar A. Tibarewala D. (2014). Automatic feature selection of motor imagery eeg signals using differential evolution and learning automata. Med. Biol. Eng. Comput. 52, 131–139. 10.1007/s11517-013-1123-9 24165805

[B2] Borghini G. Astolfi L. Vecchiato G. Mattia D. Babiloni F. (2014). Measuring neurophysiological signals in aircraft pilots and car drivers for the assessment of mental workload, fatigue and drowsiness. Neurosci. Biobeh. Rev. 44, 58–75. 10.1016/j.neubiorev.2012.10.003 23116991

[B3] Borghini G. Vecchiato G. Toppi J. Astolfi L. Maglione A. Isabella R. . (2012). Assessment of mental fatigue during car driving by using high resolution eeg activity and neurophysiologic indices. Annu. Int. Conf. IEEE Eng. Med. Biol. Soc. 2021, 6442–6445. 10.1109/EMBC.2012.6347469 23367404

[B4] Chai R. Tran Y. Naik G. R. Nguyen T. N. Ling S. H. Craig A. . (2016a). “Classification of eeg based-mental fatigue using principal component analysis and bayesian neural network,” in 2016 38th Annual International Conference of the IEEE Engineering in Medicine and Biology Society (EMBC), (Orlando, FL: IEEE), 4654–4657. 10.1109/EMBC.2016.7591765 28269312

[B5] Chai X. Wang Q. Zhao Y. Liu X. Bai O. Li Y. (2016b). Unsupervised domain adaptation techniques based on auto-encoder for non-stationary eeg-based emotion recognition. Comput. Biol. Med. 79, 205–214. 10.1016/j.compbiomed.2016.10.019 27810626

[B6] Chai X. Wang Q. Zhao Y. Liu X. Liu D. Bai O. (2018). Multi-subject subspace alignment for non-stationary eeg-based emotion recognition. Technol. Health Care 26, 327–335. 10.3233/THC-174739 29758967 PMC6004980

[B7] Chang C.-C. Lin C.-J. (2011). Libsvm: a library for support vector machines. ACM Trans. Intell. Syst. Technol. 2, 1–27. 10.1145/1961189.1961199

[B8] Charbonnier S. Roy R. N. Bonnet S. Campagne A. (2016). Eeg index for control operators mental fatigue monitoring using interactions between brain regions. Expert. Syst. Appl. 52, 91–98. 10.1016/j.eswa.2016.01.013

[B9] Chen C. Xie W. Huang W. Rong Y. Ding X. Huang Y. . (2019). “Progressive feature alignment for unsupervised domain adaptation,” in Proceedings of the IEEE/CVF Conference on Computer Vision and Pattern Recognition (Long Beach, CA: IEEE), 627–636.

[B10] Dasari D. Shou G. Ding L. (2017). Ica-derived eeg correlates to mental fatigue, effort, and workload in a realistically simulated air traffic control task. Front. Neurosci. 11:297. 10.3389/fnins.2017.00297 28611575 PMC5447707

[B11] Ganin Y. Ustinova E. Ajakan H. Germain P. Larochelle H. Laviolette F. . (2016). Domain-adversarial training of neural networks. J. Mach. Learn. Res. 17, 2096–2030. 10.5555/2946645.2946704

[B12] Hart S. G. (2006). “Nasa-task load index (nasa-tlx); 20 years later,” in Proceedings of the Human Factors and Ergonomics Society Annual Meeting (Los Angeles, CA: Sage Publications Sage CA), 904–908.

[B13] He H. Wu D. (2019). Transfer learning for brain-computer interfaces: a euclidean space data alignment approach. IEEE Trans. Biomed. Eng. 67, 399–410. 10.1109/TBME.2019.2913914 31034407

[B14] Huang X. Kortelainen J. Zhao G. Li X. Moilanen A. Seppänen T. . (2016). Multi-modal emotion analysis from facial expressions and electroencephalogram. Comput. Vis. Image Understand. 147, 114–124. 10.1016/j.cviu.2015.09.015

[B15] Hyvärinen A. Oja E. (2000). Independent component analysis: algorithms and applications. Neural Netw. 13, 411–430. 10.1016/S0893-6080(00)00026-5 10946390

[B16] Kong W. Zhou Z. Jiang B. Babiloni F. Borghini G. (2017). Assessment of driving fatigue based on intra/inter-region phase synchronization. Neurocomputing 219, 474–482. 10.1016/j.neucom.2016.09.057

[B17] Lan Z. Sourina O. Wang L. Scherer R. Müller-Putz G. R. (2018). Domain adaptation techniques for eeg-based emotion recognition: a comparative study on two public datasets. IEEE Trans. Cogn. Dev. Syst. 11, 85–94. 10.1109/TCDS.2018.2826840

[B18] Lawhern V. J. Solon A. J. Waytowich N. R. Gordon S. M. Hung C. P. Lance B. J. (2018). Eegnet: a compact convolutional neural network for eeg-based brain-computer interfaces. J. Neural Eng. 15, 056013. 10.1088/1741-2552/aace8c 29932424

[B19] Li J. Qiu S. Du C. Wang Y. He H. (2019a). Domain adaptation for eeg emotion recognition based on latent representation similarity. IEEE Trans. Cogn. Dev. Syst. 12, 344–353. 10.1109/TCDS.2019.2949306

[B20] Li J. Qiu S. Shen Y.-Y. Liu C.-L. He H. (2019b). Multisource transfer learning for cross-subject eeg emotion recognition. IEEE Trans. Cybern. 50, 3281–3293. 10.1109/TCYB.2019.2904052 30932860

[B21] Li L. (2010). “The differences among eyes-closed, eyes-open and attention states: An eeg study,” in 2010 6th International Conference on Wireless Communications Networking and Mobile Computing (WiCOM) (hengdu: IEEE), 1–4.

[B22] Liang Y. Ma Y. (2020). Calibrating eeg features in motor imagery classification tasks with a small amount of current data using multisource fusion transfer learning. Biomed. Signal Proc. Control. 62:102101. 10.1016/j.bspc.2020.102101

[B23] Liu Y. Lan Z. Cui J. Sourina O. Müller-Wittig W. (2020). Inter-subject transfer learning for eeg-based mental fatigue recognition. Adv. Eng. Inform. 46:101157. 10.1016/j.aei.2020.101157

[B24] Lotte F. Bougrain L. Cichocki A. Clerc M. Congedo M. Rakotomamonjy A. . (2018). A review of classification algorithms for eeg-based brain-computer interfaces: a 10 year update. J. Neural Eng. 15, 031005. 10.1088/1741-2552/aab2f2 29488902

[B25] Maglione A. Borghini G. Aricò P. Borgia F. Graziani I. Colosimo A. . (2014). Evaluation of the workload and drowsiness during car driving by using high resolution eeg activity and neurophysiologic indices. Annu. Int. Conf. IEEE Eng. Med. Biol. Soc. 2014, 6238–6241. 10.1109/EMBC.2014.6945054 25571422

[B26] Martin R. (2001). Noise power spectral density estimation based on optimal smoothing and minimum statistics. IEEE Trans. Speech Audio Proc. 9, 504–512. 10.1109/89.928915

[B27] Monteiro T. G. Skourup C. Zhang H. (2019). Using eeg for mental fatigue assessment: a comprehensive look into the current state of the art. IEEE Trans. Hum. Mach. Syst. 49, 599–610. 10.1109/THMS.2019.2938156

[B28] Peng X. Bai Q. Xia X. Huang Z. Saenko K. Wang B. (2019). “Moment matching for multi-source domain adaptation,” in Proceedings of the IEEE/CVF International Conference on Computer Vision (Seoul: IEEE), 1406–1415.

[B29] Polikar R. (2012). “Ensemble learning,” in Ensemble Machine Learning, eds C. Zhang and Y. Ma (Boston, MA: Springer), 1–34.

[B30] Raghu S. Sriraam N. Temel Y. Rao S. V. Kubben P. L. (2020). Eeg based multi-class seizure type classification using convolutional neural network and transfer learning. Neural Netw. 124, 202–212. 10.1016/j.neunet.2020.01.017 32018158

[B31] Saito K. Watanabe K. Ushiku Y. Harada T. (2018). “Maximum classifier discrepancy for unsupervised domain adaptation,” in Proceedings of the IEEE Conference on Computer Vision and Pattern Recognition (Salt Lake City, UT), 3723–3732.

[B32] Shore J. Johnson R. (1980). Axiomatic derivation of the principle of maximum entropy and the principle of minimum cross-entropy. IEEE Trans. Inform. Theory 26, 26–37. 10.1109/TIT.1980.1056144

[B33] Simon M. Schmidt E. A. Kincses W. E. Fritzsche M. Bruns A. Aufmuth C. . (2011). Eeg alpha spindle measures as indicators of driver fatigue under real traffic conditions. Clin. Neurophysiol. 122, 1168–1178. 10.1016/j.clinph.2010.10.044 21333592

[B34] Subha D. P. Joseph P. K. Acharya R. Lim C. M. (2010). Eeg signal analysis: a survey. J. Med. Syst. 34, 195–212. 10.1007/s10916-008-9231-z 20433058

[B35] Tzeng E. Hoffman J. Zhang N. Saenko K. Darrell T. (2014). Deep domain confusion: maximizing for domain invariance. arXiv 1412.3474.

[B36] Vecchiato G. Borghini G. Aricò P. Graziani I. Maglione A. G. Cherubino P. . (2016). Investigation of the effect of eeg-bci on the simultaneous execution of flight simulation and attentional tasks. Med. Biol. Eng. Comput. 54, 1503–1513. 10.1007/s11517-015-1420-6 26645694

[B37] Wan Z. Yang R. Huang M. Zeng N. Liu X. (2021). A review on transfer learning in eeg signal analysis. Neurocomputing 421, 1–14. 10.1016/j.neucom.2020.09.017

[B38] Wang C. Mahadevan S. (2011). “Heterogeneous domain adaptation using manifold alignment,” in IJCAI Proceedings International Joint Conference on Artificial Intelligence, Vol. 22 (Barcelona, Catalonia), 1541.

[B39] Wang G. Sun J. Ma J. Xu K. Gu J. (2014). Sentiment classification: The contribution of ensemble learning. Decis Support Syst. 57, 77–93. 10.1016/j.dss.2013.08.002

[B40] Xu G. Shen X. Chen S. Zong Y. Zhang C. Yue H. . (2019). A deep transfer convolutional neural network framework for eeg signal classification. IEEE Access 7, 112767–112776. 10.1109/ACCESS.2019.2930958

[B41] Xu R. Zhang C. He F. Zhao X. Qi H. Zhou P. . (2018). How physical activities affect mental fatigue based on eeg energy, connectivity, and complexity. Front. Neurol. 9:915. 10.3389/fneur.2018.00915 30429822 PMC6220083

[B42] Yan H. Ding Y. Li P. Wang Q. Xu Y. Zuo W. (2017). “Mind the class weight bias: weighted maximum mean discrepancy for unsupervised domain adaptation,” in Proceedings of the IEEE Conference on Computer Vision and Pattern Recognition (Honolulu, HI: IEEE), 2272–2281.

[B43] Zeng H. Dai G. Kong W. Chen F. Wang L. (2017). A novel nonlinear dynamic method for stroke rehabilitation effect evaluation using eeg. IEEE Trans. Neural Syst. Rehabil. Eng. 25, 2488–2497. 10.1109/TNSRE.2017.2744664 28858806

[B44] Zeng H. Yang C. Zhang H. Wu Z. Zhang J. Dai G. . (2019). A lightgbm-based eeg analysis method for driver mental states classification. Comput. Intell. Neurosci. 2019:3761203. 10.1155/2019/3761203 31611912 PMC6755292

[B45] Zhang B. Wang W. Xiao Y. Xiao S. Chen S. Chen S. . (2020a). Cross-subject seizure detection in eegs using deep transfer learning. Comput. Math. Methods Med. 2020:7902072. 10.1155/2020/7902072 32454884 PMC7231423

[B46] Zhang J. Yao R. Ge W. Gao J. (2020b). Orthogonal convolutional neural networks for automatic sleep stage classification based on single-channel eeg. Comput. Methods Programs Biomed. 183:105089. 10.1016/j.cmpb.2019.105089 31586788

[B47] Zhang R. Zong Q. Dou L. Zhao X. Tang Y. Li Z. (2021). Hybrid deep neural network using transfer learning for eeg motor imagery decoding. Biomed. Signal Process Control. 63:102144. 10.1016/j.bspc.2020.102144

[B48] Zhu Y. Zhuang F. Wang D. (2019). Aligning domain-specific distribution and classifier for cross-domain classification from multiple sources. Proc. AAAI Conf. Artif. Intell. 33, 5989–5996. 10.1609/aaai.v33i01.33015989

[B49] Zhu Y. Zhuang F. Wang J. Ke G. Chen J. Bian J. . (2020). Deep subdomain adaptation network for image classification. IEEE Trans. Neural Netw. Learn. Syst. 32, 1713–1722. 10.1109/TNNLS.2020.2988928 32365037

